# Results of the ARROW survey of anti-reflux practice in the United Kingdom

**DOI:** 10.1093/dote/doad021

**Published:** 2023-04-05

**Authors:** The ARROW Study Group, Natalie S Blencowe, Andrew Currie, John M Findlay, Marianne Hollyman, Steve Hornby, Phil Ireland, Shameen Jaunoo, Renol Koshy, Megan Lloyd, Anantha Mahadevan, Sheraz R Markar, Fergus Noble, Robert O’Neill, Saqib Rahman, Tim Underwood, Robert Walker, Tom Wiggins, Michael Wilson, Writing Group, Robert Walker, Andrew Currie, Tom Wiggins, Sheraz R Markar, Natalie S Blencowe, Tim Underwood, Marianne Hollyman

**Affiliations:** Guys and St Thomas’ Oesophago-Gastric Centre, Guy's & St Thomas' NHS Foundation Trust, London, UK; Faculty of Medicine, School of Cancer Sciences, University of Southampton, Southampton, UK; Service de Chirurgie Digestive A Pôle Digestif, CHU de Montpellier, Montpellier, France; Department of Bariatric Surgery, University Hospitals Birmingham, Birmingham, UK; Department of Molecular Medicine and Surgery, Karolinska Institutet, Karolinska University Hospital, Stockholm, Sweden; Nuffield Department of Surgery, University of Oxford, Oxford, UK; Population Health Sciences, University of Bristol, Bristol, UK; Division of Surgery, Head and Neck, University Hospitals Bristol NHS Foundation Trust, Bristol, UK; Faculty of Medicine, School of Cancer Sciences, University of Southampton, Southampton, UK; Upper Gastrointestinal Surgery Department, Musgrove Park Hospital, Taunton, UK

**Keywords:** acid reflux, anti-reflux surgery, fundoplication, gastro-esophageal reflux (GERD), GORD, surgery

## Abstract

Gastro-esophageal reflux disease (GERD) is a common, significant health burden. United Kingdom guidance states that surgery should be considered for patients with a diagnosis of GERD not suitable for long-term acid suppression. There is no consensus on many aspects of patient pathways and optimal surgical technique, and an absence of information on how patients are currently selected for surgery. Further detail on the delivery of anti-reflux surgery (ARS) is required. A United Kingdom-wide survey was designed to gather surgeon opinion regarding pre-, peri- and post-operative practice of ARS. Responses were received from 155 surgeons at 57 institutions. Most agreed that endoscopy (99%), 24-hour pH monitoring (83%) and esophageal manometry (83%) were essential investigations prior to surgery. Of 57 units, 30 (53%) had access to a multidisciplinary team to discuss cases; case-loads were higher in those units (median 50 vs. 30, P < 0.024). The most popular form of fundoplication was a Nissen posterior 360° (75% of surgeons), followed by a posterior 270° Toupet (48%). Only seven surgeons stated they had no upper limit of body mass index prior to surgery. A total of 46% of respondents maintain a database of their practice and less than a fifth routinely record quality of life scores before (19%) or after (14%) surgery. While there are areas of consensus, a lack of evidence to support workup, intervention and outcome evaluation is reflected in the variability of practice. ARS patients are not receiving the same level of evidence-based care as other patient groups.

## INTRODUCTION

Gastro-esophageal reflux disease (GERD) is defined as a condition that develops when the reflux of stomach contents causes troublesome symptoms and/or complications.^1^ Excluded from this definition is gastrointestinal pathology that is not reflux but may have some overlapping features such as gastric volvulus or para-esophageal hernia.

GERD has a worldwide prevalence of up to one-in-three adults^2^ and conveys a significant healthcare burden.^3^ For many, optimal therapy is provided by lifestyle modifications and proton pump inhibitors (PPI). However, some have persistent reflux or do not wish to take medication and desire further interventions.^4^ Anti-reflux surgery (ARS) offers effective control for severe GERD, but can have adverse effects.^5^^,^^6^ Current guidance from the National Institute of Health and Care Excellence (NICE) states that ARS should be considered for patients with a confirmed diagnosis of acid reflux and who are not suitable for, or do not wish long-term acid suppression therapy.^7^

Although there have been recent recommendations in pre-operative workup from the British Society of Gastroenterology (BSG),^8^ and the International Consensus Regarding Pre-operative Examinations and Clinical Characteristics Assessment to Select Adult Patients for Anti-reflux Surgery (ICARUS) guidelines,^9^ there is a lack of clarity in many aspects of the patient pathway and a lack of consensus regarding the optimal ARS technique.^10^ Technical uncertainties include the extent of dissection (i.e. hiatal dissection and division of short gastric vessels^11^), fundoplication formation (i.e. partial, full, anterior or posterior^12–14^), whether gastropexy is required^15^ and the method of crural repair^16^ (including the use of mesh reinforcement^17^). Alternative minimally invasive ARS techniques such as LINX™,^18^^,^^19^ Stretta™^20^^,^^21^ and EsophyX™^20^^,^^22^ are available, although how they fit into GERD treatment algorithms remains unclear.

A previous study has highlighted significant variation in England in the provision of ARS, although clinical outcomes were comparable.^23^ A further study looking at reintervention rates showed a 9.4% reoperation rate and 59.5% continued PPI rate.^24^ Risk factors for reoperation and PPI use included female sex and increasing age but the study lacked the resolution required to better inform patient selection. There is an absence of information on how patients are currently being selected in clinical practice and further granular detail on the delivery of ARS is required. To address this, a United Kingdom-wide, prospective database was designed to record current practice in ARS for 1 year.^25^ As the first step, the current study was designed to survey surgeon opinion regarding pre-, peri- and post-operative practice.

## METHODS

This study has been reported according to the Checklist for Reporting Results of Internet E-Surveys (CHERRIES).^26^ The study protocol was published in Diseases of The Esophagus and provides details of the methodology.^25^ We defined GERD according to the Montreal classification as a condition which develops when the reflux of stomach contents causes troublesome symptoms and/or complications. We excluded emergency interventions for giant para-esophageal hernia as these cases do not meet the definition of GERD. Survey questions were iteratively developed by the Audit and Review of anti-Reflux Operations and Workup (ARROW) steering committee. The final questionnaire consisted of 90 fields per surgeon and 57 fields per institution.^25^ Participants were enlisted through AUGIS (Association of Upper Gastrointestinal Surgeons), ROUX group of Upper GI surgical trainees and social media. An online tool (https://www.aleaclinical.eu) was adapted and developed to collect data and piloted in three centers prior to launch. The survey was circulated in November 2019 and closed in March 2020. Estimates given therefore reflect pre-COVID-19 practice. Explorative comparisons of responses were undertaken according to funding type [National Health Service (NHS) and private practice] presence of an ARS multidisciplinary team (MDT), physiology access, surgeon expertise and practice volume.

### Data analysis

Proportions were compared using the Chi squared test unless otherwise stated. Medians were compared using the Wilcoxon rank sum test for two groups or the Kruskal-Wallis analysis of variance for three or more groups. Correlations were assessed using the Pearson correlation coefficient. Results were considered significant if *P* < 0.05. All data are available on request.

## RESULTS

Survey responses were received from 155 surgeons at 57 institutions across the United Kingdom with a median of 40 cases per institution per year (range 10–200) and four surgeons per institution (range 1–8) **(**Supplementary Table 1**)**. Approximately half of respondents performed malignant esophago-gastric surgery 76/155 (49%) and 57 performed bariatric surgery (37%); just 13 surgeons performed both as part of their normal practice and 36 performed neither (38.6%) (Table 1**)**.

**Table 1:** Scope of practice, investigations required and procedures offered by the respondents to the survey

Number of Cases Performed Annually	Median (range{interquartile range})	
NHS	12 (0–75{10–20})	
Private	6 (0–75{4–15})	
		
Primary Practice	**n/155 (%)**	
Benign upper gastrointestinal (GI) (total)	133 (85.8%)	
No Bariatric, No resectional (total)	36 (23.2%)	
Bariatric (total)	57 (36.8%)	
Esophago-gastric (EG) resectional (total)	76 (49.0%)	
Hepatopancreaticobiliary (HPB) (total)	2 (1.3%)	
EG resectional and Bariatric	13 (8.4%)	
EG resectional and HPB Resectional	1 (0.6%)	
		
What investigations do you consider compulsory prior to ARS?	**n/155 (%)**	
EGD	153 (98.7%)	
24-hour pH monitoring	128 (82.6%)	
Any resolution manometry	130 (82.8%)	
Standard resolution manometry	103 (65.6%)	
High resolution manometry	27 (17.4%)	
Upper GI contrast study	33 (21.3%)	
24-hour impedance monitoring	15 (9.7%)	
Wireless pH monitoring (BRAVO)	4 (2.6%)	
Computed Tomography (CT)	4 (2.6%)	
		
Procedures offered	**NHS n/155 (%)**	**Private n/48(%)**
Fundoplication	154 (98.1%)	45 (93.8%)
LINX	7 (4.5%)	9 (18.8%)
Stretta	4 (2.5%)	5 (10.4%)
Roux-en-Y gastric bypass	49 (31.2%)	16 (33.3%)

ARS, anti-reflux surgery; NHS, National Health Service.

**Fig. 1:**
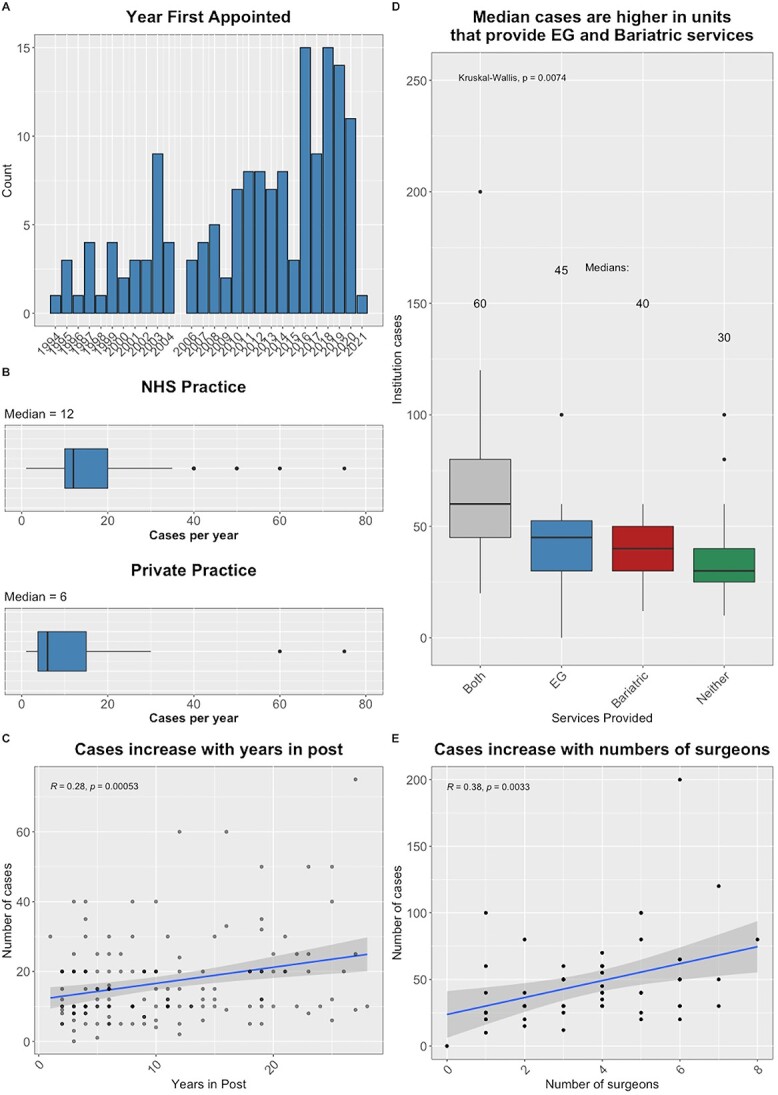
Characteristics of survey respondents. (A) Year first appointed as consultant. (B) Cases performed in NHS and private practice. (C) Case-volume increased with years in post. (D) Median cases were higher in centers that had either sub-specialist services on site. (E) Cases increased with the number of surgeons. (NHS, National Health Service).

Surgeons had a median of 7 years’ Consultant experience **(**Fig. 1A**),** with a range of 1–26 years. The median number of cases performed per year, by individual surgeons in the NHS was 12 (range 0–75) (Fig. 1B**)**. There was a trend to higher volume with increasing experience (Fig. 1C). In total, 48 surgeons (31%) also performed ARS in the private sector, where median cases per year were six (range 0–75) (Table 1). There was no link between sub-specialization and volume of ARS on an individual surgeon level but units with bariatric or esophago-gastric cancer services performed higher volumes of ARS overall, and units with a higher number of surgeons tended to perform more ARS overall **(**Fig. 1D). Case-volume also correlated positively with years in post (Fig. 1E).

Surgeons had to apply for funding on an individual patient basis at two institutions; despite the extra step, these centers maintained a high volume of operations (70 and 80 case per year). All units had access to EGD and upper gastrointestinal contrast studies (UGCS) for the assessment of GERD. Esophageal physiology studies were provided internally at 75% of NHS Trusts, externally within the NHS at 22% and in private laboratories at 2 trusts. (Supplementary Table 1). On-site physiology services had no effect on patient volume.

### Pre-operative assessment

Most surgeons (99%) agreed that EGD, 24-hour pH monitoring (83%) and esophageal manometry (83%) were essential investigations prior to ARS**.** Just over a fifth (21.3%) of respondents believed that an UGCS was essential **(**Table 1**)**.

**Fig. 2:**
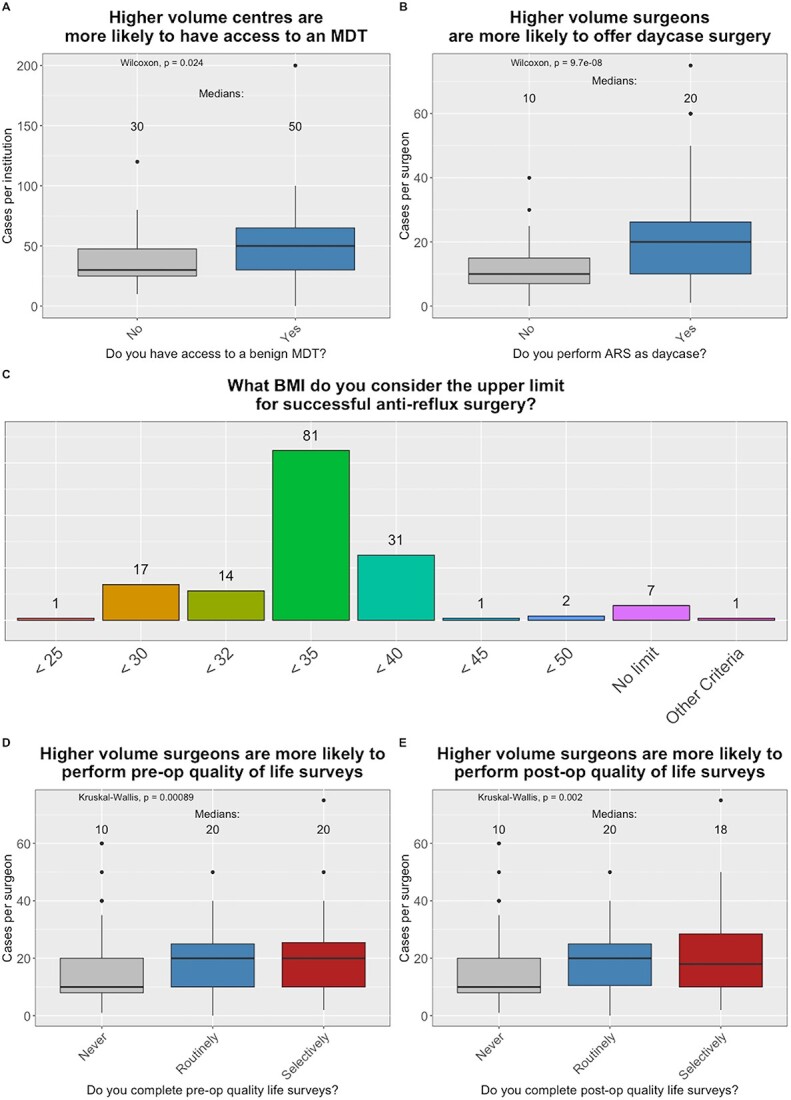
Relationship between case-volume and workup. (A) Centers with access to a benign MDT had higher case-loads. (B) Surgeons offering day-case surgery had higher median case-loads. (C) The upper limit of BMI that participants believe results in successful ARS. (D) and (E) Surgeons who assessed the pre-and post-operative quality of life had higher case-volumes. (ARS, anti-reflux surgery; BMI, body mass index; MDT, multidisciplinary team).

Of the 57 units surveyed, 30 (53%) had access to a MDT to discuss ARS cases; case-loads were higher in units with access to an MDT (median 50 vs. 30, *P* < 0.024) **(**Fig. 2A**)**. In total, 10 of these units routinely discussed all ARS patients in MDT, 17 in selected cases and in three institutions MDT discussion was reserved for revisional surgery. Regional MDTs were available for five institutions and the remainder were locally held. All MDTs were attended by surgeons and 73% included gastroenterologists. Radiologists were available in 60% of MDTs; specialist nurses and physiologists were only present in a minority (43 and 37%, respectively) of MDTs. Pathologists, respiratory physicians, dieticians and Ear, Nose and Throat (ENT) surgeons were also reported to attend MDTs at individual units.

### Surgical techniques

Almost all surgeons (98%) offered a form of fundoplication with a third also offering Roux-en-Y gastric bypass as ARS (Table 1). In both the NHS and private practice, there was limited use of novel interventions such as LINX™ and STRETTA™.

When reporting technical aspects of fundoplication, the most popular fundoplication type was a Nissen posterior 360° offered by 115 (75%) surgeons, followed by a posterior 270° Toupet (48%) (Table 2). The most popular anterior fundoplication was a Dor 180° (40%). There were no significant differences in the type of fundoplication performed between NHS and private practice. Most surgeons (69%) selected the fundoplication according to symptoms, manometry findings or both. Most surgeons (84%) reported they either routinely or selectively divide the short gastric vessels during fundoplication. Around a quarter of surgeons report using bougies to calibrate the sizing of the fundoplication. For hiatal repair, most routinely perform a posterior cruroplasty. Anterior cruroplasty was used selectively by most, but 10% of respondents stated they would never undertake this. A total of ˂20% of surgeons report using Collis esophageal lengthening procedures, even selectively. Of 36 units where technique information was available for more than one surgeon only two units (of five and two surgeons) had a ‘uniform’ approach to surgery and the remaining units all demonstrated ‘non-uniform’ surgical approaches and preferences within the same unit.

**Table 2:** Surgical techniques employed for fundoplication

Procedures offered	NHS	Private
Nissen posterior 360^o^	115 (74.2%)	31 (64.6%)
Toupet posterior 270^o^	74 (47.7%)	26 (54.2%)
Toupet posterior 180^o^	18 (11.6%)	8 (16.7%)
Dor anterior 180^o^	62 (40.0%)	18 (37.5%)
Watson anterior 180^o^	40 (25.8%)	10 (20.8%)
Partial anterior 90^o^	1 (0.6%)	1 (2.1%)
Collis	8 (4.5%)	2 (4.2%)
Other	0(0.0%)	2 (4.2%)
		
Do you tailor the type of wrap for individual patients?		
Yes, based on clinical symptoms	35 (22.6%)	
Yes, based on manometry findings	72 (46.5%)	
Never	48 (31.0%)	
		
Do you divide the short gastric arteries?		
Routinely	69 (44.5%)	
Selectively	61 (39.4%)	
Never	25 (16.1%)	
		
Do you perform an anterior cruroplasty?		
Routinely	18 (11.6%)	
Selectively	121(78.1%)	
Never	16 (10.3%)	
		
Do you perform a posterior cruroplasty?		
Routinely	138 (89.0%)	
Selectively	13 (8.4%)	
Never	4 (2.6%)	
		
Do you perform a Collis gastroplasty?		
Routinely	1 (0.6%)	
Selectively	27 (17.4%)	
Never	127 (81.9%)	
		
Do you size the wrap with a bougie?		
Routinely	23 (14.8%)	
Selectively	14 (9%)	
Never	118 (76.1%)	

NHS, National Health Service.

### Post-operative management and investigations

Just over half (52%) **(**Table 3**)** of respondents offered some form of ARS as a day-case either in the NHS or private practice. There was no difference in the likelihood of providing day-case surgery in the NHS or private sector. Surgeons offering day-case procedures had higher median cases per year (20 vs. 10, *P* < 0.001) **(**Fig. 2B**)**. Tolerance of liquid diet was required prior to same day discharge for 84% of respondents and 15% required patients to tolerate a solids. Other important factors influencing day-case discharge were proximity of patients’ residence (44%) and the time-of-day surgery was completed (54%).

**Table 3:** Procedures offered as day-case (length of stay <24 hours), and criteria used

Procedures offered as day-case	NHS n/155 (%)	Private n/48(%)
Fundoplication	76 (49.0%)	21 (43.8%)
LINX	10 (6.5%)	9 (18.8%)
Stretta	4 (2.5%)	6 (12.5%)
Roux-en-Y gastric bypass	1 (0.6%)	0 (33.3%)
None	75 (48.4%)	17 (35.2%)
		
Criteria applied for day-case discharge	**n/80 (%)**	
Tolerance of solid oral intake	12 (15.0%)	
Tolerance of liquid oral intake	67 (83.8%)	
Proximity of patient residence from hospital	35 (43.8.%)	
Surgery completed by a specific time	43 (53.8%)	
Pain Controlled	3 (3.8%)	
Age	2 (2.5%)	
Availability for telephone follow-up	1 (1.3%)	
		
Do you discharge patients with an antiemetic?	**n/155 (%)**	
Routinely	69 (44.5%)	
Selectively	61 (39.4%)	
Never	25 (16.1%)	
		
Do you discharge patients with an opioid analgesia?	**n/155 (%)**	
Routinely	18 (11.6%)	
Selectively	121(78.1%)	
Never	16 (10.3%)	
		
Do you record pre-op symptom severity/QoL scores?	**n/155 (%)**	
Routinely	29 (18.7%)	
Selectively	28 (18.1%)	
Never	98 (63.2%)	
		
Do you record post-op symptom severity/QoL scores?	**n/155 (%)**	
Routinely	22 (14.2%)	
Selectively	30 (19.4%)	
Never	103 (76.1%)	

QoL, quality of life scores.

Nearly half (45%) of respondents routinely prescribed antiemetics and 39% utilized these in selected cases. Routine use of opioid analgesia was rare (12%).

Most institutions offered in-person clinic appointments with a surgeon as standard follow-up [49/57 (86%)] (Supplementary Table 1). Routine use of post-operative investigations was rare with only eight surgeons performing a routine post-op EGD and 12 surgeons performing a routine upper GI contrast swallow.

### Anti-reflux surgery and obesity

Just over 60% of surgeons believed that ARS can be effective in patients with obesity but only seven surgeons stated they had no upper limit of body mass index (BMI) for non-gastric bypass ARS. The most utilized upper BMI limit for consideration of non-gastric bypass ARS was 35 kg/m^2^ which was utilized by 52% of surgeons (81/155) (Fig. 2C). However, there was wide variation in specific BMI criteria for non-gastric bypass ARS.

A total of 116 (75%) surgeons either routinely (57%) or selectively (14%) request patients with obesity complete a pre-operative low-calorie diet to facilitate liver shrinkage. However, there was considerable variation regarding the criteria to apply prior to this request. Some surgeons set BMI criteria of 32, 35 or 40 kg/m^2^ before requiring a pre-operative diet. Others relied solely on clinical suspicion or clinical findings (such as presence of central obesity), whereas 70% applied no specific criteria.

### Data monitoring

Less than half (46%) of respondents maintain a database of their ARS practice and less than a fifth routinely record symptom severity or quality of life scores (QoL) before (19%) or after (14%) surgery. The 71 (46%) surgeons who keep a database had higher case-loads (median 10 vs. 20 cases *P* = <0.001). The 62 (40%) surgeons who record QoL scores before or after surgery also had higher case-loads **(**Fig. 2D and E).

## DISCUSSION

This study has highlighted in several areas there is good conformity in practice; in other areas we demonstrate the broad variation in ARS practice and opinion across the United Kingdom. This reflects the lack of high-quality evidence to determine provision required to support good clinical practice. The ICARUS,^9^ BSG^8^ and NICE guidelines^7^ provide recommendations to support the assessment and selection of patients for ARS. However, of the 37 statements assessed in the ICARUS Delphi process, only one statement ‘Patients with heartburn as the main symptom who respond satisfactorily to proton pump inhibitors (PPIs) are good candidates for antireflux surgery’ was considered to have high-quality supporting evidence. Similarly in the 27 BSG guidelines statements only five were considered to have high grade evidence to support them. Of these, four out of the five statements referred to technical aspects of performing manometry studies rather than their interpretation in the clinic. Faced with a lack of evidence, it is no surprise that clinical practice is so varied. It is a widely held belief that each fundoplication technique has its benefits and drawbacks^10^ and therefore alternatives are constantly being proposed and developed. However, we demonstrate an absence of a patient-tailored approach in many surgeons, suggesting that the reality is that surgeons develop a preference and then stick to that preference.

Units demonstrated variation in case-volume between centers and surgeons. Those with onsite physiology, MDT access and a greater number of surgeons had higher case-volumes. Centers that provide ARS as a day-case have higher case-volumes and centers with bariatric or esophago-gastric cancer services had higher case-volumes. Evidence to support either centralization of services or continued practice in low-volume centers is lacking. Just over half of centers make use of MDTs to discuss ARS, with some services making full use of MDTs and others never using an MDT. No one has attempted to demonstrate the benefit of MDT assessment prior to ARS and yet in some centers with higher case-loads it is routine while in other centers patients are denied any potential benefit; further work should be done to assess the potential benefit to patients.

While around half of surgeons maintain a database of operations, less than a fifth record pre-operative severity and even fewer record the effects of their intervention on symptoms. This represents a void in knowledge about how well we are serving patients. The surgical community in the United Kingdom will address this with the ARROW audit and subsequent launch of the National Hiatal Surgery Registry, both under the guidance of AUGIS.

Patients with a BMI >35 kg/m^2^ would not be offered ARS by more than half of surgeons but in some centers a BMI of >30 kg/m^2^ would be considered too high, and in others no limit was set. This suggests a postcode lottery for access to ARS for the obese. NICE,^8^^,^^9^^,^^27^ BSG and ICARUS guidelines do not set recommendations on obesity in ARS. In fact the ICARUS guidelines specifically state that morbid obesity should not be a barrier to ARS citing three recent studies.^28–30^ Two subsequent meta-analyses have shown that BMI >30 kg/m^2^ is associated with increased operative time and increased risk of failure^31^^,^^32^ although the authors stopped short of recommending against surgery.

There is no core outcome set (COS) for ARS. Some centers report using GERD-Q,^33^ and a triple assessment using Reflux Severity Index (RSI-9),^34^ Eating Assessment Tool (EAT-10)^35^ and Voice Handicap Index (VHI-10)^36^ tools were used by some survey participants. The absence of a COS with validated criteria valued by patients makes audit and quality assurance difficult.

This is the first nationwide survey of practice in the United Kingdom. It does have some limitations: data are self-reported, from a subset of the population and not possible to cross-validate patient volumes per center and surgeon sub-specialty. We excluded emergency hiatal surgery such as obstructed or ischaemic giant para-esophageal hernias from this survey, and they are excluded from the ARROW audit. This is due to the absence of existing standards and guidelines to audit against and the subsequent ethical considerations. This, however, represents a patient group managed by expert hiatal surgeons in the centers surveyed here. Emergency hiatal surgery can therefore be considered a similarly under-researched and under-monitored area of sub-specialty practice.

ARS patients are not receiving the same level of evidence-based care as other patient groups such as cancer patients. The evidence to support workup, intervention, personalization and outcome comparisons is lacking. Yet GERD patients can be expected to live longer with the consequences of decision making. Large centers with EG cancer and bariatric services have more surgeons, perform more ARS, are more likely to have access to an MDT and are more likely to monitor pre- and post-surgery patient QoL.

The ARROW prospective audit will establish current practice, compliance with clinical guidelines and inform COSs, improvement projects and randomized trials in the future.

### Local Collaborators

Frank Curran, John Spearman, Nilanjana Tewari, Alex Boddy, Sukhbir Ubhi, David Exon, David Bowrey, Christopher Sutton, Ashutosh Tandon, Nitin Arvind, James Hewes, Christopher Wong, James Hopkins, Robert Williams, Owain Jones, Will Hawkins, Ewen Griffiths, Borys Darmas, Andrew Robertson, Barry Dent, Sanjay Taribagil, Vijay, John Bennett, Andrew Hindmarsh, Richard Hardwick, Peter Safranek, Oliver Old, Marianne Hollyman, Paul Marriott, Syed Iftikhar, Natasha Henley, Ashok Menon, Paul Leeder, James Byrne, Nick Maynard, Michael Wilson, Richard Morgan, Paul Glen, Stefan Antonowicz, A Martinez-Isla, Duncan Beardsmore, Vittal Rao, Richard Thompson, Osama Elhardello, Nima Abbassi-Ghadi, Pritam Singh, V Charalampakis, John Findlay, Paul Super, David Khoo, Dipankar Mukherjee, Samrat Mukherjee, Thangadorai Amalesh, Spyridon Kapoulas, Nick Davies, Richard Byrom, David Bennett, Khaleel Fareed, Lauren Kennedy Brian Stewart, Nagammapudar Balaji, Alistar Sharples Kanagaraj Marimuthu, Brijesh Madhok, Altaf Awan, George Bouras, Oliver Priest, Rishi Singhal, David Mahon, Aruna Munasinghe, Olga Tucker, Saj Wajed, Steve Hornby, Simon Dwerryhouse, Christopher Peters, Anupam Dixit, Anthony Perry, Gijs van Boxel, Paul Goldsmith, Matthew Mason, Peter Sedman, Richard Skipworth, Chetan Parmar, Richard Krysztopik, Richard Welbourn, Dimitri Pournaras, Antonios Athanasiou, Andreas Luhmann, Alasdair MacMillan, Richard Newton, Chris Pring, Richard Bowyer, Chandra Cheruvu, Stuart Andrews, Andrew Cockbain, Nicholas Boyle, Nathan Howes, Christopher Grocock, Nathan Stephens, Mark Hartley, Ben Byrne, John Loy, Vamshi Jagadesham, Rohan Gunasekera,Rohith Gopala Rao, George Kirby, Yogesh Kumar Pratik, A Sufi, Khaled Dawas, Nicholas Johnson, Ashish Rohatgi, Bhaskar Kumar, Michael Lewis, Edward Cheong, Loveena Sreedharan, Manoj Kumar, Natasha Ross, Shay Nanthakumaran, Clive Kelty, Peter Mekhail, Arin Saha Mark Peter, Rob Adair, Tamir Salih, Peter Driscoll, Alex Reece-Smith, Samer Humadi, Shashi Irukulla, Kumaran Ratnasingham, Neville Menezes, James Gossage, Andrew Davies, Mansoor Khan, John Benett, Vijay Sujendran, Andrew Crumley, Liz Cannings, Martin Wadley, Ben Knight, Euan Mclaughlin, YKS Viswananth, Philip Pucher, Mr David Griffith, David Hewin, Waleed Al-Khyatt, Hamish Noble, Brian Dobbins, Andrew Cowie.

## FUNDING

This project is funded by support provided by the Royal College of Surgeons and is part funded by the Association of Upper Gastrointestinal Surgeons and Heartburn Cancer UK.

## Supplementary Material

Supplementary_table_1_doad021

## Notes

Data Analysis: Robert Walker

## Notes

Steering Committee: Natalie S. Blencowe, Andrew Currie, John M. Findlay, Marianne Hollyman, Steve Hornby, Phil Ireland, Shameen Jaunoo, Renol Koshy, Megan Lloyd, Anantha Mahadevan, Sheraz R. Markar, Fergus Noble, Robert O’Neill, Saqib Rahman, Tim Underwood, Robert Walker, Tom Wiggins, Michael Wilson.

## Notes

Design: Multicenter survey of current clinical practice.
