# Supplementary material for: Results of the ARROW survey of anti-reflux practice in the United Kingdom
Source: Dis Esophagus. 2023 Apr 5;36(10):doad021. doi: 10.1093/dote/doad021 (PMC10543366; doi:10.1093/dote/doad021)
Supplement: Supplementary_table_1_doad021 [file supplementary_table_1_doad021.docx]

Supplementary table 1: Institutional survey results

| Number of Surgeons | median(range,{interquartile range}) |
| --- | --- |
|  | 4 (1-8,{2-5}) |
|  |  |
| Number of Cases Performed Annually | **median(range,{interquartile range})** |
|  | 40 (10-200,{30-60}) |
|  |  |
| Institution Practice | **n/57 (%)** |
| Benign upper GI | 57 (100%) |
| Bariatric | 27 (47.4%) |
| EG resectional | 25 (43.9%) |
| EG resectional and Bariatric | 17 (29.8%) |
| Benign upper GI alone | 22 (38.6%) |
|  |  |
| Available Investigations | **n/57 (%)** |
| EGD | 57 (100%) |
| 24-hour pH monitoring | 27 (47.4%) |
| Any resolution manometry | 53 (93.0%) |
| Standard resolution manometry | 25 (43.9.%) |
| High resolution manometry | 50 (87.7%) |
| Upper GI contrast study | 57 (100%) |
| 24-hour impedance monitoring | 37 (64.9%) |
| Wireless pH monitoring (BRAVO) | 18 (31.6%) |
| CT | 55 (96.5%) |
|  |  |
| Manometry Availability | **n/57 (%)** |
| NHS lab within own Trust | 43 (75.4%) |
| NHS lab at another Trust | 13 (22.8%) |
| Private lab | 2 (3.5%) |
|  |  |
| Unit standardisation | **n/57 (%)** |
| Does your institution have a standardised pre-op information sheet? | 41 (71.9%) |
| Does your institution have a standardised post-op diet sheet followed by all surgeons? | 45 (78.9%) |
|  |  |
| What is your institution’s routine follow up arrangement? | **n/57 (%)** |
| In-person clinic appointment with doctor | 49 (86.0%) |
| Telephone clinic (doctor) | 5 (8.8%) |
| Telephone clinic (nurse) | 2 (3.5%) |
| None | 1 (1.8%) |
